# Evaluation of a Deep Learning and XAI based Facial Phenotyping Tool for Genetic Syndromes: A Clinical User Study

**DOI:** 10.1101/2025.06.08.25328588

**Published:** 2025-06-09

**Authors:** Ömer Sümer, Tobias Huber, Dat Duong, Suzanna E. Ledgister Hanchard, Cristina Conati, Elisabeth André, Benjamin D Solomon, Rebekah L. Waikel

**Affiliations:** 1 Human-Centered Artificial Intelligence, University of Augsburg, Augsburg, Germany; 2 Technische Hochschule Ingolstadt, Ingolstadt, Germany; 3 Medical Genetics Branch, National Human Genome Research Institute, Bethesda, MD, USA; 4 Department of Computer Science, University of British Columbia, BC, Canada

**Keywords:** artificial intelligence, deep learning, explainability, decision support, dysmorphology

## Abstract

Artificial intelligence (AI) tools are increasingly employed in clinical genetics to assist in diagnosing genetic conditions by assessing photographs of patients. For medical uses of AI, explainable AI (XAI) methods offer a promising approach by providing interpretable outputs, such as saliency maps and region relevance visualizations. XAI has been discussed as important for regulatory purposes and to enable clinicians to better understand how AI tools work in practice. However, the real-world effects of XAI on clinician performance, confidence, and trust remain underexplored. This study involved a web-based user experiment with 31 medical geneticists to assess the impact of AI-only diagnostic assistance compared to XAI-supported diagnostics. Participants were randomly assigned to either group and completed diagnostic tasks with 18 facial images of individuals with known genetic syndromes and unaffected individuals, before and after experiencing the AI outputs. The results show that both AI-only and XAI approaches improved diagnostic accuracy and clinician confidence. The effects varied according to the accuracy of AI predictions and the clarity of syndromic features (sample difficulty). While AI support was viewed positively, users approached XAI with skepticism. Interestingly, we found a positive correlation between diagnostic improvement and XAI intervention. Although XAI support did not significantly enhance overall performance relative to AI alone, it prompted users to critically evaluate images with false predictions and influenced their confidence levels. These findings highlight the complexities of trust, perceived usefulness, and interpretability in AI-assisted diagnostics, with important implications for developing and implementing clinical decision-support tools in facial phenotyping for rare genetic diseases.

## INTRODUCTION

While rare diseases collectively affect nearly 1 in 10 individuals, individual rare diseases affect fewer than 1 in 2000 (according to European definitions). Most rare diseases have genetic causes, and most are extremely uncommon. For example, the prevalence of 22q11.2 deletion syndrome (22qDS) is approximately 1 in 4,000 live births^1^, while Kabuki syndrome (KS) affects approximately 1 in 32,000^2,3^. The rarity of these conditions and the large number of rare diseases (more than 7000) makes diagnosis challenging.

Deep learning (DL), a subset of artificial intelligence (AI), has advanced biomedical research, including genetic disease diagnostics^4^. AI-driven tools can be used to analyze facial images to assess the differential diagnosis by detecting dysmorphic features. However, conventional deep learning architectures, such as convolutional neural networks, lack inherent human-explainable decision-making processes. Further, explainability has emerged as a key factor in the regulation of medical devices and algorithms involving AI. Efforts to improve interpretability have led to the use of techniques like saliency maps, which highlight key image regions influencing AI predictions^5–7^. Despite their widespread adoption, the practical utility of these explainable AI (XAI) approaches remains uncertain.

In clinical practice, medical experts increasingly use AI-based tools for various tasks despite disparities in users’ comfort, experience, and trust^8^. Though suggested as necessary by some regulatory bodies, clinicians, and researchers, it is not yet clear how helpful XAI approaches may be in practice. For example, it remains uncertain whether saliency maps could effectively assist clinicians in their tasks or enhance their trust and understanding of the AI systems. Developing better XAI systems is essential to bridge the gap between AI’s opaque decision-making and the need for clinical validation. Ensuring that AI-generated insights are interpretable, trustworthy, and verifiable by medical professionals is in turn critical for their integration into clinical practice.

Previous studies have suggested that AI tools may not improve human diagnostic reasoning; for instance, although large language models (LLMs) outperform physicians, LLMs fail to enhance their decision-making^9^. Overall, results of AI-based clinical decision support systems have been mixed; a systematic review found only “sparse evidence” that the use of such systems is associated with improved clinician performance at diagnostic tasks.^10^ Such data and examples underscore the complexity of human-AI collaboration, difficulties in assessing effects, and the pressing need to investigate the interplay between AI performance, clinician trust, and diagnostic behavior in many instances^11^, including AI-assisted facial phenotyping in medical genetics.

In a previous study^12^, we explored saliency maps and region-based explanations for AI-driven facial phenotyping of genetic conditions with visible syndromic facial features. Our region-based explanations aggregate the relevance of pixels within specific regions, such as the eyes or mouth, to simplify human interpretation of the explanation. In our current study, we conducted a web-based survey to assess the real-life impact of AI-only and XAI support on clinical diagnosis tasks. We use the term AI-only to denote an AI system where the prediction outputs are provided without any further explanations.

## OBJECTIVES

This study examines how medical geneticists, particularly those with expertise in facial dysmorphology, utilize and are influenced by AI-generated predictions and XAI in their diagnostic decision-making. In other words, the central question is whether the use of DL and XAI may successfully fulfil the “fundamental theorem” of informatics^13^. According to this theorem, we wanted to test whether an information resource (DL results and XAI) could improve human (in this case medical geneticists) performance with a task (recognition of genetic conditions). In this study, we aimed to evaluate the effects of the DL results and XAI in a controlled simulation prior to embarking on more complex and expensive “real-world” prospective studies.^14^

To test this, we solicited board-certified or board-eligible medical professionals specializing in medical genetics; a total of 18 participants completed the AI-supported diagnosis task, while 13 participated in the XAI-supported group. Specifically, we assess whether AI-only and XAI improved participant performance and explore key factors influencing this effect, including the accuracy of AI model predictions and the visibility of clinical features in facial images. Beyond performance, we evaluated how AI and XAI interventions affect participants’ confidence, as model predictions can reinforce or undermine trust. Notably, we expected incorrect AI predictions and explanations of these predictions to lead users to approach results with greater skepticism.

Understanding how medical geneticists assess facial dysmorphology and perceive AI-driven outputs is critical for improving human-AI collaboration. These insights will not only inform the development of AI/ML-based Software as a Medical Device (SaMD) for facial phenotyping but also have the potential to significantly enhance diagnostic accuracy and clinical utility in future healthcare applications.

## MATERIALS AND METHODS

We trained a convolutional neural network model (ResNet-50) to classify face images as one of the five genetic conditions or unaffected (see section 2.1). For each image, the model provides prediction probability for each of the labels. Then, we computed the corresponding DeepLIFT saliency maps^15^ and region relevance scores as in Sümer, et al.^12^. These AI-only (e.g., providing just prediction probabilities) and XAI outputs (e.g., providing prediction probabilities and explanations) will be used in our user survey. The below section will outline the data collection and participant recruitment processes. Following this, we will describe the methodology used to compare the diagnostic task performance of medical geneticists with the predictions of the AI model and the impact of XAI output interventions. Finally, we will detail the statistical methods employed to compare the intervention groups.

### Data collection and image selection

Following our previous work, we used publicly available facial images of individuals with 22qDS (OMIM# 611867), Angelman syndrome (AS) (OMIM# 105830), KS (OMIM# 147920, 300867), Noonan syndrome (NS) (OMIM# 163950, 605275, 609942, 610733, 611553, 613706, 615355, 616559, 616564, 619087), and Williams syndrome (WS) (OMIM# 194050), as well as images of unaffected individuals. These five syndromes were selected due to their recognizable facial features, the variable degree of difficulty in identification based on facial features (e.g., the features of NS may be more obvious and easier to recognize than those of 22qDS), and because they are common enough for most practicing medical geneticists to have first-hand experience with affected individuals.

A total of 3547 images were included in the full dataset: 22qDS (n=591), AS (n=456), NS (n=329), KS (n=247), WS (n=529), unaffected individuals (n=228), and other genetic syndromes (n=1167). Available metadata included age, sex, and ancestry. Eighteen images (three per syndrome and three unaffected) were selected for the user study. These images represented pediatric individuals (newborn to 18 years), varying ancestries, and both sexes. Three clinical geneticists independently reviewed the images to confirm that they accurately depicted one of the five syndromes or an unaffected individual (that is, they would be considered to show a relatively typical presentation of the represented conditions).

Furthermore, all eighteen images were unseen test cases, not included in the training dataset of the AI models. This ensured that the AI model’s performance in the user study reflected its real-world predictive capabilities. For use in the experiments, we intentionally selected images that were classified correctly (2 images per syndrome) and incorrectly (one image per syndrome) by the AI model, preserving a balanced test case scenario to evaluate both AI and XAI support.

### Recruitment of Participants

Medical geneticists, particularly those with expertise in facial dysmorphology, were recruited for this study. Participants were identified through professional networks and publications in the field. Recruitment was conducted via email, providing participants with a link to one of two surveys corresponding to the AI-only or XAI intervention (see descriptions below). Of 124 international medical geneticists invited, 28 completed the survey, and 8 partially completed it. Partially completed surveys were included in the analysis if they completed the diagnosis task for more than half of the 18 face images. The study was approved as IRB-exempt by the NIH IRB (IRB# 002178).

Participants in this study were all board-certified or board-eligible medical professionals who self-identified as medical geneticists. The majority in both the baseline and XAI groups had more than 10 years of clinical experience, with most affiliated with academic medical or research centers. Given the random assignment of participants, both groups reported similar distributions in prior knowledge and professional background, suggesting comparability in assessing the effects of AI and XAI support (See Supplementary Figure 1 and Supplementary Table 1 for participants’ years of experience in genetic diseases and distribution of their institutional affiliations.)

### Comparison of survey interventions.

Each survey included three images of different individuals with each of the five syndromes and three unaffected images for a total of 18 images for each participant to classify. The task was to view these face images and answer a diagnosis task to pick one of the five genetic syndromes or unaffected. Participants were not told prior to the survey which type of intervention they would receive nor the number of images of each type.

We compared two types of interventions via surveys sent using Qualtrics (Provo, Utah, United States). Surveys were specific to one of two survey interventions:

- AI-only (baseline) group: test images + prediction output
- XAI group: test images + prediction + XAI tool outputs

Both survey versions included a tutorial about how AI-based classification generates a prediction probability (the percent likelihood an individual has a particular genetic syndrome) and a description of each of the five genetic syndromes, including a reference image. The XAI tool survey version also included a tutorial about our XAI methods, including information about the saliency maps and the region relevance outputs. Participants were shown each of the 18 images one at a time, initially without any intervention. For each image, they were asked to classify the image as one of the five syndromes or ”unaffected” and rate their confidence level in their classification. They were then shown the image a second time with the AI classifier’s prediction probability with or without the XAI tool outputs and were asked again to classify the image and rate their confidence level. In addition to the classification tasks, participants provided demographic information and answered questions about their use and opinions of AI diagnostic tools.

**Figure. 1:**
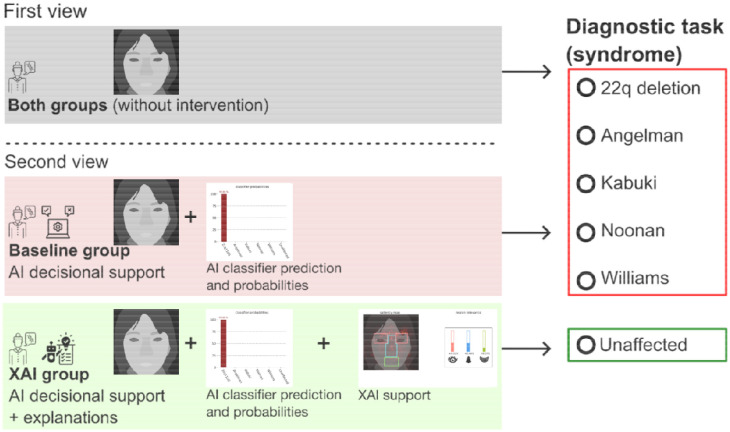
Overview of the AI- and XAI-assisted diagnostic task workflow. In the first view, participants from both groups examined facial images without assistance and selected a diagnosis from five genetic conditions or ”unaffected,” while also rating their confidence. In the second view, participants received AI support: the Baseline group saw AI predictions and probabilities, while the XAI group additionally received two explainability features: saliency maps and region relevance scores. After, both groups repeated the diagnostic task, reassessed their confidence, and rated the usefulness of the provided support. Note: Real facial images were used in the survey. To compile with medRxiv guidelines, real facial images have been replaced with segmentation representations of a face of an individual with a given genetic condition.

Saliency maps are one of the widely used methods in explainable AI. They calculate the importance of input features for the decision of an AI model by either using a model’s internal mechanisms and parameters (model specific) or are independent of the AI model (model agnostic). We used DeepLIFT^5^, which calculates the relevance of neurons between consecutive layers of a neural network. By automatically aligning face images, we create masks around the eyes, nose, and mouth area and calculate a measure of region relevance score as depicted in Figure 1. A more detailed evaluation of different saliency map algorithms is available in our previous work^12^.

In this user study of medical geneticists, we investigated the impact of AI and XAI support on diagnostic performance and user perceptions. Specifically, we investigated whether AI and XAI assistance influence diagnostic accuracy, including the effect of AI model accuracy (true vs. false predictions) on diagnostic outcomes and potential variations across different genetic conditions. Additionally, we examine whether AI and XAI support enhance users’ self-rated confidence in their diagnoses.

Beyond performance and confidence, we explored how users perceive the usefulness of AI-generated predictions and XAI explanations, such as saliency maps and region relevance. We further assessed whether perceived usefulness correlates with diagnostic improvement following AI/XAI support. Finally, we analyzed qualitative user feedback to derive additional insights into the role of AI and XAI in clinical decision-making.

### Evaluation metrics and statistical analysis

Participants in both the AI-only and XAI groups initially viewed the facial images and completed a diagnostic task (selecting the most likely genetic syndrome or an `ùnaffected” option) without AI or XAI assistance, and subsequently rating their confidence on a five-point Likert scale. In the second viewing, each group reviewed the same images accompanied by AI-generated outputs. The participants then repeated the diagnostic task and provided confidence ratings. Finally, they assessed the AI tool’s helpfulness across three aspects (prediction probabilities, saliency maps, and region relevance representations) using a five-point Likert scale.

This experimental design enabled a comparison of three key measures before and after the intervention in both groups:

- Performance improvement was assessed by comparing diagnostic accuracy between the first and second views, with possible outcomes of −1 (True→False), 0 (True→True or False→False), or +1 (False→True). In other words, if the participants change their previously correct answer to a wrong choice, then the outcome is −1. Conversely, the outcome would be +1 if the participants changed their previously incorrect answer to the correct answer. When participants’ answers for the two views stayed either correct or incorrect (whether or not their initial choice was correct or incorrect), then the outcome score is 0.
- Confidence improvement was quantified as the difference in self-reported confidence scores, ranging from −2 (“not confident”) to +2 (“highly confident”), between the first and second views (i.e., the view without XAI guidance vs. the one with XAI guidance).
- Usefulness of AI and XAI support was only rated after the second viewing on a Likert scale ranging from −2 (“not useful”) to +2 (“very useful”). The AI-only group evaluated only the helpfulness of seeing just the prediction probabilities. In contrast, the XAI group assessed the usefulness of seeing both the prediction probabilities and the saliency map and region relevance explanations.

We compared prediction and confidence improvements between baseline and XAI groups before and after the intervention, reporting mean values and confidence intervals (1.06 times the unbiased standard error of the mean). These measures were analyzed by aggregating participant-level data, particularly in two situations: (i) when AI model predictions are true or false, and (ii) across the groups of pictures containing more obvious clinical features and challenging ones with dysmorphic features more difficult to notice. Statistical testing was conducted only to compare these measures. For hypothesis testing between the baseline and XAI groups, such as differences in prediction improvement, we first evaluated normality and equality of variance using the Shapiro-Wilk and Levene’s tests. Based on these results, either an independent t-test or a Mann-Whitney U-test was applied as appropriate.

Since the baseline group did not have usefulness ratings for explanations, we did not compare these ratings between groups. Instead, we conducted a correlation analysis to examine the relationship between participants’ perceived usefulness of each component and prediction improvement. Given that both variables are ordinal and do not follow a normal distribution, we used Spearman’s correlation, which assesses whether greater prediction improvement is associated with higher perceived usefulness, even in the absence of a strictly linear relationship. This analysis relies on two key assumptions: (1) each test image is approximately independent and exchangeable, and (2) participants have comparable experience with genetic conditions. These assumptions allow us to relate the aggregated usefulness ratings and prediction improvement ratios within each group.

Various factors may influence participants’ confidence and trust in AI and XAI interventions beyond those captured by diagnostic tasks, confidence ratings, and usefulness assessments. To explore these additional factors, we collected open-ended feedback at the end of the survey. A qualitative analysis of these responses highlighted potential strengths and limitations of AI-assisted facial phenotyping, providing insights to guide future research.

## RESULTS

### Impact of AI- vs. XAI-assisted decision-making diagnostic performance

We compared the overall prediction improvement in both the AI-only and XAI groups before and after the intervention. The mean prediction improvement was slightly lower in the XAI group (0.031± 0.129) compared to the AI-only baseline (0.056± 0.11). The means are similar enough and the standard deviations are large and, thus we investigated other factors.

**Figure 2:**
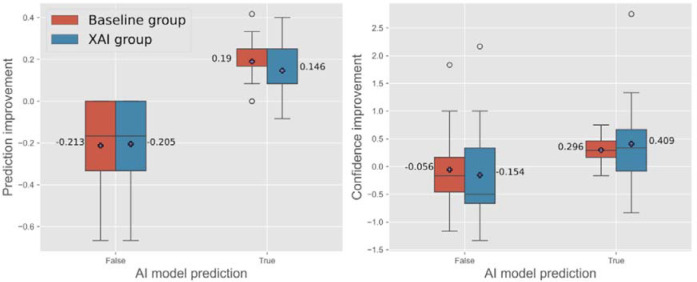
Prediction improvement (left) and confidence improvement (right) are shown for cases where the AI model’s predictions were true and false. In both Baseline and XAI groups, interventions positively impacted participants’ prediction performance when the AI model was correct. However, when the AI model made false predictions, participants’ performance and confidence decreased. While prediction improvement was similar across these cases, XAI interventions led to lower confidence (−0.154) in falsely predicted samples, suggesting that participants were more critical of the AI’s predictions and less likely to be misled.

Figure 2 (left side) illustrates prediction improvement in both groups, stratified by the accuracy of the AI model’s prediction (true vs. false). When the AI model prediction was correct, the AI-only baseline (0.190± 0.120) exhibited greater prediction improvement than the XAI group (0.146±0.120); however, due to large standard deviations, this difference was not statistically significant (*t*(29) = 1.07, r=.19, ns). Conversely, when the AI model prediction was incorrect, both the XAI group (−0.205±0.256) and the baseline group (−0.213±0.234) were negatively affected.

**Figure 3:**
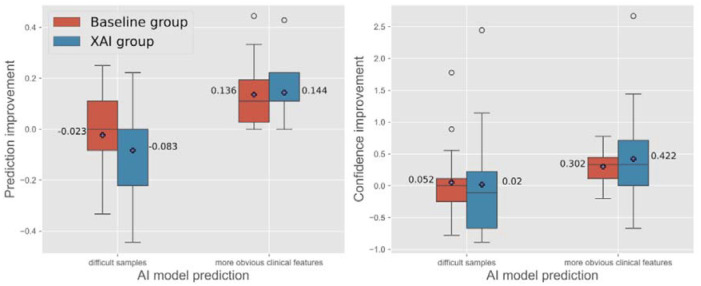
Prediction improvement (left) and confidence improvement (right) are grouped by cases where more obvious clinical features are present; easier to decern for clinicians (9 images), versus more challenging samples with increased difficulty (9 images). All values are aggregated per participant. Greater positive change in both groups’ prediction performance and confidence ratings was observed in the group of images with more obvious clinical features.

Another factor that may affect participants’ responses is whether there are more obvious dysmorphic features. Figure 3 (left side) shows the prediction improvement across sample difficulty. Two independent clinicians categorized facial images into two equally-sized image sets (n = 9 per set): one set with more easily identifiable clinical features and another with more subtle features. Prediction improvement of difficult images was an average of −0.023±0.162 in AI-only and −0.083±0.208 in XAI groups, respectively. For the images with more obvious clinical features, the AI-only group’s average prediction improvement was 0.136±0.126 and 0.144±0.115 for the XAI group.

Conditioned on the AI-only participants, easy images accuracy average (0.136±0.126) have significantly better prediction improvement than hard images (−0.023±0.162), *U* = 246.5, p < .01. Similarly, for the XAI participants, their prediction improvement median among easy images (0.144±0.115) are greater than hard images (−0.083±0.208), *U* = 138.0, p < .01. Based on both analyses, it appears that in the cases where AI model predictions were false, or images had subtle, difficult to recognize features, both the AI-only baseline and the XAI intervention tended to reduce prediction improvement.

### Impact of AI- vs. XAI-assisted decision-making on user confidence

Beyond diagnostic performance, we examined changes in user confidence. As shown in Figure 2 (right side), XAI support appeared to enhance average confidence scores in correctly predicted images as compared to AI only (0.409 vs.0.296). However, in cases where the AI prediction was incorrect, confidence decreased more in the XAI group (−0.154) compared to the AI-only baseline group (−0.056). Moving to the distribution of confidence improvement with respect to sample difficulty, the participants rated their confidence higher in easy images vs. difficult images in both AI-only (0.302 vs.0.052) and XAI (0.422 vs. 0.02) groups (Figure 3, right side). In the AI-only group, confidence improvement for easy images (0.333±0.257) is significantly greater than the one in difficult images (0±0.557), U = 245.0, p .01. However, in the XAI group, confidence ratings for the easy images (0.333±0.846) and difficult images (−0.111±0.904) did not significantly differ from each other, *U* = 115.5, p= 0.12.

### Perceived usefulness of AI model output and explanations

**Figure 4:**
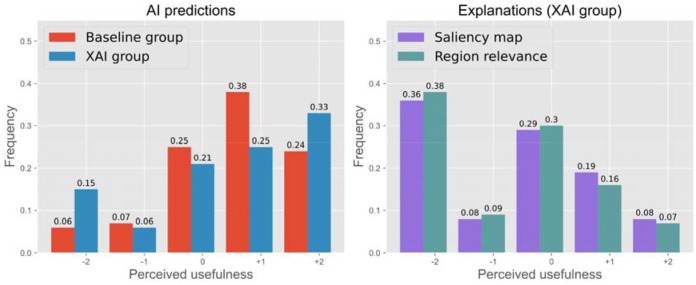
Participants’ perceived usefulness of AI predictions (left), saliency maps, and region relevance scores(right). Overall, respondents in both the Baseline and XAI groups found AI predictions more useful than saliency maps and region relevance scores. In the XAI group, the ratings of saliency maps and region relevance scores were nearly identical.

Overall, the participants appreciated seeing the AI predicted probabilities, regardless of whether these participants picked the correct answers, nor whether these predictions were accurate (roughly positive trend in the Figure 4A histogram and Supplementary Table 2). The usefulness scores of seeing the AI predictions among the AI-only and XAI participants on average are 0.660±1.102, and 0.543±1.391, respectively. The reverse trend is seen with the saliency maps and region relevance score usefulness (Figure 4B). On average, the XAI responses yielded negative scores for these two metrics −0.443±1.362 and −0.552±1.333, respectively. We note that Figure 4B histogram appears bimodal, where participants either strictly viewed XAI as unhelpful, or they generally had a neutral perception. For example, over 1/3 of participants (36% and 38% of the participants) selected a −2 score (the lowest score possible) for saliency maps and relevance scores usefulness (Figure 4B, left side), but the perception of the remaining participants is more normally distributed with mean close to 0. This finding is also reflected in the open-ended user feedbacks in Supplementary Table 2.

Next, conditioned on the user improvement, Table 1 measures the correlation between this outcome and the perceived usefulness of seeing the AI predictions, the saliency maps, and the region relevance scores. Interestingly, seeing the AI predictions did not correlate with user improvement (although the users broadly appreciated seeing the AI predictions (Figure 4A)). Only saliency maps and region explanations demonstrated weak but statistically significant correlations between usefulness ratings and user improvement (*rho* = .151 and .166, respectively). Furthermore, a strong and significant correlation (*rho* = .927) existed between the user usefulness ratings for the saliency maps and the region relevance explanations. Finally, ignoring the user improvement, there are correlations between usefulness scores of seeing the AI predictions with both the saliency maps and region relevance scores (rho = 0.375 and 0.167, respectively) (Table 1). Thus, even though explanation methods did not improve user accuracy, they correlate well with how useful the AI predictions would be perceived.

### Users’ Qualitative Feedback on AI and XAI Support

In addition to determining whether there was a qualitative difference between AI and XAI support, we also sought to determine attitudes and opinions of these interventions. At the end of the survey, we included an optional open-ended question asking each participant to provide feedback on the support tools they used during the survey. The majority of participants included feedback (13/18 and 11/13 for the AI-only and XAI groups, respectively) (see Supplementary Table 2 and respective word cloud representations in Supplementary Figure 5).

For this question, we asked participants to reflect upon how much they relied on the tools to decide which condition images showed. To quantify the results, key words and phrases were identified to determine whether participants found the AI tools helpful. Of the 13 participants in the AI-only survey, 9 reported that the AI tool was helpful in their diagnoses, whereas 3 reported it was not helpful, and 1 provided a neutral comment. In contrast, of the 11 participants in the XAI survey, only 3 reported the tools were helpful, and 7 reported they were not helpful, with 1 reported a neutral response. While a description of how to use XAI tools was included in the introductory part of the survey, 2 XAI survey participants reported not knowing how to use the tools.

## DISCUSSION

### Impact of AI and XAI support on diagnostic performance

#### AI improved diagnostic performance when it was correct

With respect to accuracy, similar trends were seen for both AI-only and XAI participants. When AI predictions were accurate, then participants were more likely to switch their initial incorrect answer into the correct answer in the second viewing (Figure 2). In contrast, false AI predictions either had no significant effect or mislead participants into picking an incorrect answer in the second viewing. We suspect that if a participant initially picked an accurate answer but possibly lacked familiarity with the diseases, then this participant might have been misled by an incorrect AI prediction in the second viewing. In such a situation, XAI could be particularly valuable since the users can inspect the areas-of-interests as determined by the AI classifier. Indeed, on average, the participants had lower confidence when seeing incorrect AI predictions (−0.056 vs −0.154 for AI and XAI) and vice versa with correct AI predictions (0.296 vs 0.409 for AI and XAI) (Figure 2B). However, the standard errors are large, and future study (e.g., recruiting more participants) would be needed to fully verify this hypothesis.

**Figure 5:**
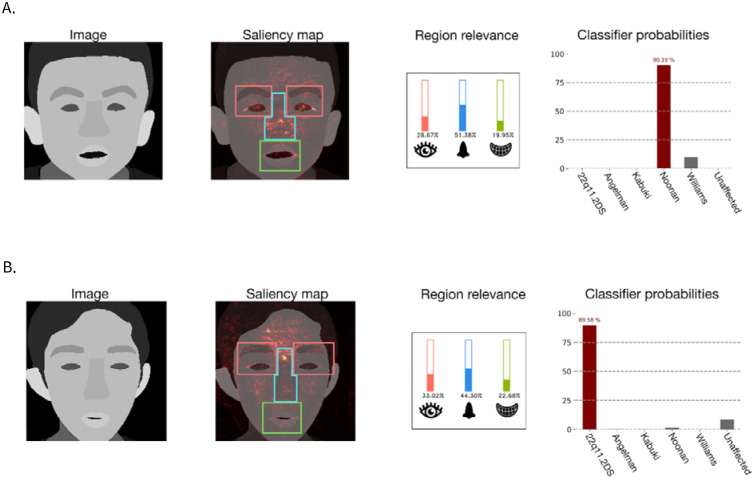
Examples of AI model predictions, saliency maps and region relevance score histograms. An example of the AI model’s accurate prediction of an individual with Noonan syndrome is shown in panel A. An example of the AI model’s inaccurate prediction of an individual with Kabuki syndrome; falsely predicted as 22q11.2 deletion syndrome is shown in panel B. Note: Real facial images were used in the survey. To compile with medRxiv guidelines, real facial images have been replaced with segmentation representations of a face of an individual with a given genetic condition.

One possible reason for AI intervention’s negative impact on falsely predicted cases is the model prediction probabilities. For example, an image showing a person with KS was incorrectly predicted to be 22qDS with a prediction probability of 89.5% (Figure 5B). This overconfidence may mislead users, reinforcing incorrect diagnoses.

Another factor influencing AI intervention effectiveness is the obviousness of dysmorphic features in the images. Our results show that participants were more likely to revise their diagnoses correctly following AI or XAI intervention when clinical features were more apparent. Moreover, sample difficulty may also affect both the AI classifier and the quality of explanations. When a classifier struggles to detect subtle dysmorphic features, the saliency estimation methods may similarly fail to highlight small anomalies.

#### XAI did not significantly enhance user performance and perception

On average, the XAI intervention did not lead to a higher performance improvement compared to the AI-only cohort. Due to the nature of our study, participants were required to decide even when they were unconvinced by the explanations and would have normally sought additional patient information in a real clinical setting. Conversely, XAI might have made the participants more critical of the disease predictions (discussed in subsequent sections). While carefully-controlled experiments can be efficient and cost-effective, these observations highlight the fact that caution is warranted when attempting to extrapolate results from a study like this to real-world settings^14^.

Nevertheless, our findings are consistent with previous studies of clinical approaches, which also did not show significant improvements from saliency maps in objective tasks^16,17^. Similar to our low perceived usefulness ratings and the negative textual feedback regarding saliency maps, those studies also found that saliency maps were difficult to interpret and increased users’ cognitive load. Other XAI-assisted strategies may be more intuitive for users. For instance, Mertes et al. found counterfactual explanations to be significantly more useful than saliency maps in a pneumonia classification task^17^. However, it is important to note that their task is considerably simpler than diagnosing rare genetic conditions based on facial images. Therefore, a specific future study would be needed to see confirm whether counterfactuals are also more helpful in identifying rare genetic diseases. As an additional note of caution, while there may be themes that hold true across different clinical XAI studies, it is also possible that there are highly specific nuances that need to be considered in the context of different medical areas. In a previous study, we identified specific patterns by which experts and non-experts assessed the faces of individuals with suspected genetic conditions^18^. This “human pattern” of assessing faces – which appears to distinct from saliency map results – may not be relevant to the assessment of other types of clinical images, like X-rays or pathology images. That is, the findings in this analysis may be highly important to medical genetics, where clinical diagnosis often depends on the facial appearance. The results may generalize to assessing photos of people with other genetic conditions in addition to the ones selected for study but may be less generalizable to tasks in other medical domains^10^.

### Other comparisons

#### Participants did not seem to differentiate saliency maps and region relevance

We found a strong correlation between the usefulness ratings for the saliency maps and the region relevance explanations. This finding indicates that the participants did not differentiate between the region relevance and saliency maps. Furthermore, in the free-text feedback of the participants, they used both terms interchangeably.

#### Example images

To gain deeper insights into the potential benefits and risks of XAI methods, we examined two images that showed a strong difference in prediction improvement between the AI-only and XAI groups (see Supplementary Figures 2 and 3). Members of our group with expertise in clinical dysmorphology provided their interpretation of these examples.

Figure 5A shows an example on an individual with Noonan syndrome where the AI made a correct prediction of Noonan syndrome. In this case, the XAI group exhibited both greater prediction improvement and higher confidence in their prediction. The medical expert provided an intuition for these findings, explaining: “*The saliency map, particularly in the eye region, helps emphasize the downslanting palpebral fissures (the angle of the eyes), which is a very characteristic/classic sign of Noonan syndrome. When I look at the saliency image, the first thing I notice is the saliency map showing the ways the eyes are positioned.”* This observation suggests that the saliency map aligns well with the AI’s prediction. Such alignment may explain why the explanation increased participants’ confidence in the AI’s correct decision.

Figure 5B shows an example of an individual with KS where the AI’s prediction incorrectly predicted 22qDS instead. In this case, both groups exhibited negative prediction improvement, but the decline was less pronounced in the AI-only baseline group. Consistent with the overall trend, the confidence in this wrong prediction was lower in the XAI condition.

The expert noted:

“I noticed that the eyebrows are noticeably highlighted. Although the eyebrows of people with Kabuki syndrome can have a characteristic appearance, this fact may not be very well known to clinicians (including the survey respondents), and this may have been distracting or led respondents to doubt the diagnosis of Kabuki syndrome. […] The image is rather difficult, I feel. It does have some features that I feel are indeed reminiscent of 22q11.2 deletion syndrome, such as the shape of the nose, including the nasal bridge. More broadly, 22q11.2 is often quite difficult to identify based on facial findings unless clinicians are very experienced with this condition.”

The second part of the expert’s comment suggests that there may be some justification for the AI’s incorrect prediction of 22qDS, which could explain why participants believed the incorrect AI output. At the same time, the highlighted eyebrows (a feature observed in KS, the correct diagnosis) may have contributed to reduced participant confidence in the wrong prediction. While only anecdotal, this example suggests that domain experts may be able to provide useful insight in designing and evaluating clinical XAI studies.

### Limitations of our study

Although this study’s findings are relevant to considerations regarding the use of XAI (and related methods) in clinical practice, we emphasize that there are limitations.

To obtain representative results, we conducted the user study with a specific type of clinician, medical geneticists. Taking time out of their busy schedules is difficult for most clinicians, and there is only a limited pool of clinicians who are familiar with rare genetic diseases. Therefore, this was a small study in terms of the number of participants, different genetic conditions included, and individual images viewed. This likely limits the generalizability of the findings.

Despite the provided explanations of survey components and sample cases, participants’ prior knowledge, biases, and familiarity with XAI may have influenced their usefulness ratings. The perceived value of XAI explanations is also closely tied to the performance of saliency methods and the classifier itself. Our prior study correlated saliency-based explanations with expert-annotated phenotypic regions (e.g., eyes, nose, and mouth), selecting DeepLIFT as the best-performing method. However, these techniques struggled to capture subtle dysmorphic features, such as those affecting the region of the nose, potentially diminishing user confidence in their reliability. Aligning saliency explanations with phenotypic features could improve their perceived usefulness. However, this points to another open question: Does more perceived usefulness also lead to better prediction improvement? The explanations could mask mistakes of the classifier and mislead the user, as seen in Figure 5B. Other studies have suggested correlations between explainability, trust, and understanding, and future studies could explore these correlations further in the medical genetics context^11^.

Our user study used a single classifier based on a ResNet architecture. However, the specific classifier, training data composition, and model architecture influence the saliency maps---their highlights and overall structure. On the one hand, if the saliency maps were independent of the classifier, they would fail to provide meaningful explanations for the classifier. On the other hand, this dependency means we cannot be sure how the saliency maps would change if different classifiers were used (e.g., classifiers that use a vision transformer architecture). As a result, this paper’s findings regarding saliency maps are inherently tied to the model we employed. Further studies using diverse classifier architectures would be necessary to fully determine whether these results generalize across different classifiers.

In this paper, our image classifier is based on the ResNet architecture^12^. Besides the data training composition, the choice of model architecture can often influence the saliency maps. However, due to our small dataset, we opted for ResNet architecture instead of other larger networks such as Vision Transformer. Future studies should include collecting more data and exploring other deep learning architectures. We also note that saliency maps depend on the model’s performance; for example, when the model misclassified an image, the saliency map with respect to this ground-truth label may not accurately highlight all the syndromic features. In this case, one should not trust the saliency map^19,20^. However, the ground-truth labels are often unavailable when recognizing syndromic faces in a clinical setting. Hence, in practice, we cannot anticipate whether the model prediction is accurate; thus, inspecting the saliency maps is sensible.

There are only three main face regions (eyes, nose, and mouth) assessed in this study, but many relevant phenotypes (i.e., our previous study^12^ indicated 50 phenotypes associated with these genetic conditions). Instead of highlighting the regions, pointing out possible phenotypes (such as Human Phenotype Ontology) would better lead users to identify the most likely genetic conditions. Instead of saliency maps, one potential solution would be an alternative XAI method called concept bottleneck models^21^. Alternatively, a future study incorporating vision-language models^22^ and interactive prompting may make explanations more understandable for users.

As described above, there may also be human-specific patterns to assessing faces, and XAI studies involving faces may be affected by these patterns. As a future study, it would be interesting to compare the results across different image types used in medical genetics (such as faces, skeletal X-rays, and skin findings)^23–25^.

## CONCLUSIONS

While our AI/XAI support was perceived as useful ---especially in cases where it aligned with participants’ diagnostic reasoning---it had limited impact on overall diagnostic performance. Recent work has underscored the heterogeneous outcomes of human-AI collaboration across tasks and domains, with some scenarios showing a decline in performance when AI is introduced^26^. In the context of facial phenotyping, which remains central in medical genetics, our study suggests a potential for synergy, indicating that AI explanations can support clinicians when appropriately aligned with their diagnostic process. To build on this promise, future research should address sources of confusion and skepticism identified by users and conduct larger-scale studies to better understand the conditions under which XAI enhances diagnostic accuracy.

## Supplementary Material

1

## Figures and Tables

**Table 1:** Correlation analysis between participants’ perceived usefulness ratings and prediction improvement. Spearman’s Rho, degrees of freedom, and p-values are reported.

*Baseline group*		Prediction usefulness	Saliency usefulness	Region usefulness
Prediction improvement	Spearmans’ Rho	.041	—	—
	df	*304*	—	—
	p-value	*.480*	—	—
*XAI Group*				
Prediction improvement	Spearmans’ Rho	−.028	**.151**	**.166**
	df	*228*	*228*	228
	p-value	*.480*	*.022*	.011
Saliency usefulness	Spearmans’ Rho	**.376**	—	—
	df	*228*		
	p-value	<*001*		
Region usefulness	Spearmans’ Rho	**.167**	**.927**	—
	df	*228*	*228*	
	p-value	*.011*	<*001*	

## Data Availability

Code to train model and make saliency maps are at https://github.com/sumeromer/facial-gestalt-xai. Code to reproduce figures and statistical tests for surveys are at https://github.com/rlwaikel/FacialXAI. Raw results of the user survey responses are also available on the project repository. OS has full access to all of the data in the study and takes responsibility for the integrity of the data and the accuracy of the data analysis.
