# Supplementary material for: Evaluation of a Deep Learning and XAI based Facial Phenotyping Tool for Genetic Syndromes: A Clinical User Study
Source: medRxiv. 2025 Jun 9:2025.06.08.25328588. Preprint. [Version 1] doi: 10.1101/2025.06.08.25328588 (PMC12191099; doi:10.1101/2025.06.08.25328588)
Supplement: 1 [file NIHPP2025.06.08.25328588V1-supplement-1.pdf]

medRxiv preprint doi: https://doi.org/10.1101/2025.06.08.25328588; this version posted June 9, 2025. The copyright holder for this preprint (which was not certified by peer review) is the author/funder, who has granted medRxiv a license to display the preprint in perpetuity. This article is a US Government work. It is not subject to copyright under 17 USC 105 and is also made available for use under a CC0 license.

## SUPPLEMENTARY INFORMATION

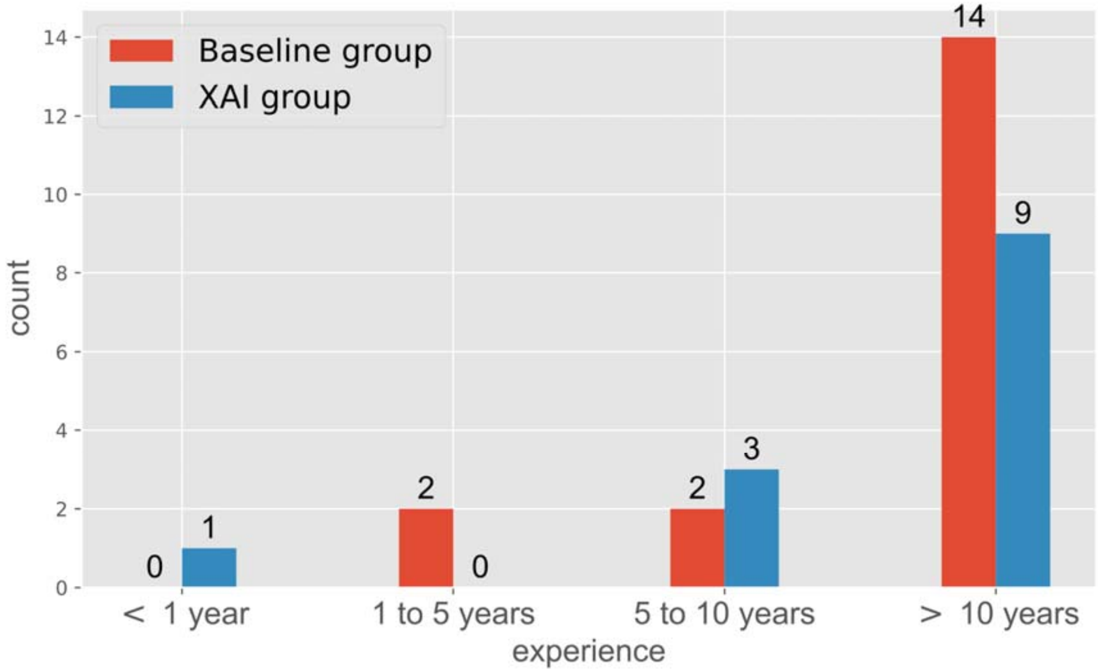


**Supplementary Figure 1**: Participants' years of experience in rare genetic diseases.

**Supplementary Table 1**: Participants' affiliated institutions in Baseline and XAI groups.

| | Baseline group | XAI group |
|---|---|---|
| Academic medical or research center | 12 | 11 |
| Community based hospital | 4 | 1 |
| Molecular diagnostic company | 1 | 1 |
| Other | 1 | 0 |

medRxiv preprint doi: https://doi.org/10.1101/2025.06.08.25328588; this version posted June 9, 2025. The copyright holder for this preprint (which was not certified by peer review) is the author/funder, who has granted medRxiv a license to display the preprint in perpetuity. This article is a US Government work. It is not subject to copyright under 17 USC 105 and is also made available for use under a CC0 license.

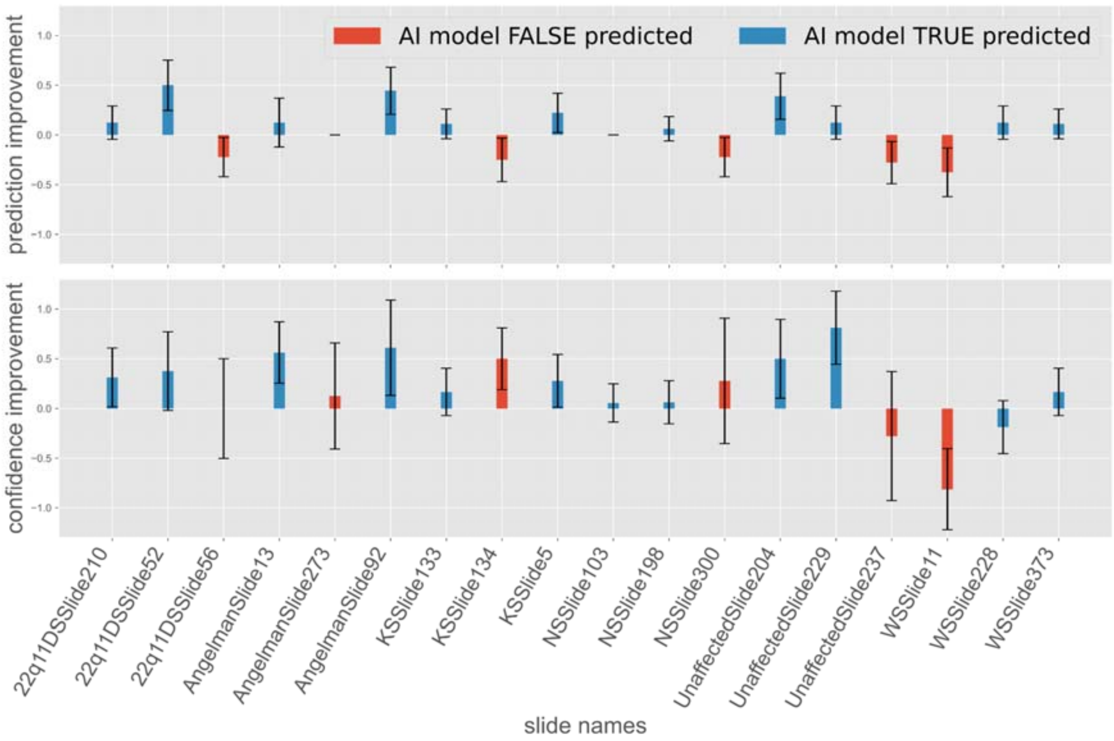


**Supplementary Figure 2**: Confidence and prediction improvement in the Baseline group, viewed separately for each image (18 images in total). Cases where the AI model's prediction was false are marked in red. The only intervention provided was the AI model's prediction and probabilities.

medRxiv preprint doi: https://doi.org/10.1101/2025.06.08.25328588; this version posted June 9, 2025. The copyright holder for this preprint
(which was not certified by peer review) is the author/funder, who has granted medRxiv a license to display the preprint in perpetuity.
This article is a US Government work. It is not subject to copyright under 17 USC 105 and is also made available
for use under a CC0 license.

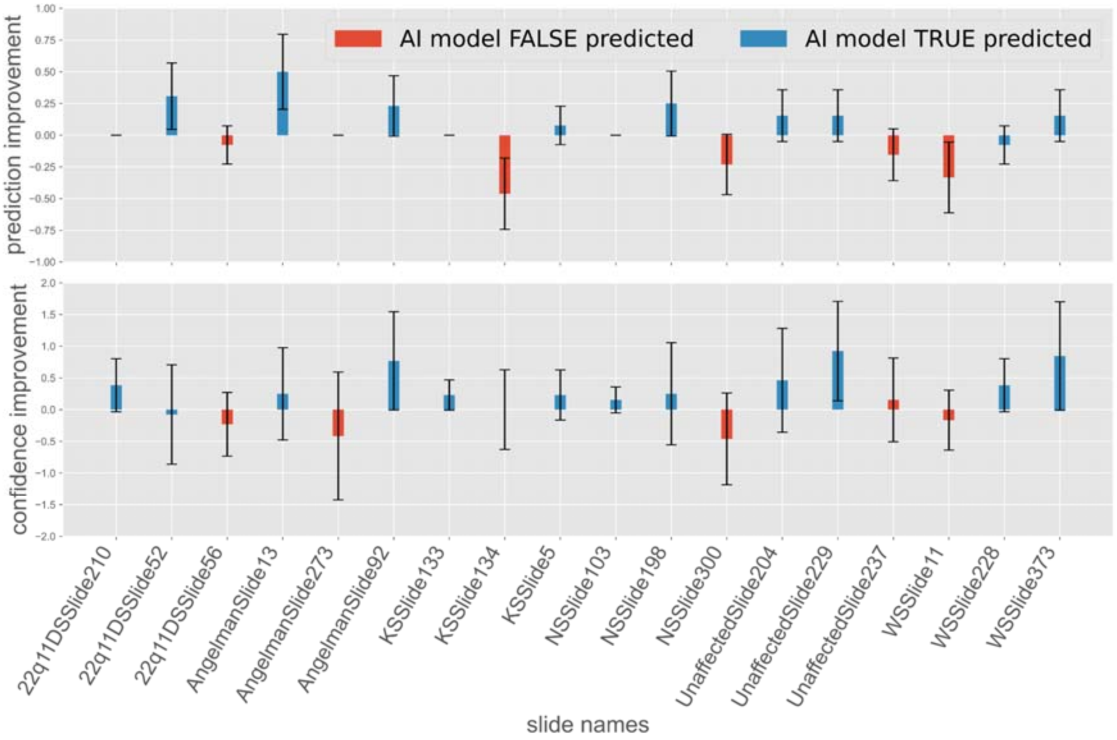


**Supplementary Figure 3**: Confidence and prediction improvement in the XAI group, viewed separately for each image (18 images in total). Cases where the AI model's prediction was false are marked in red. The intervention provided was the AI model's prediction and probabilities, saliency maps and region relevance representations.

medRxiv preprint doi: https://doi.org/10.1101/2025.06.08.25328588; this version posted June 9, 2025. The copyright holder for this preprint (which was not certified by peer review) is the author/funder, who has granted medRxiv a license to display the preprint in perpetuity.
This article is a US Government work. It is not subject to copyright under 17 USC 105 and is also made available for use under a CC0 license.

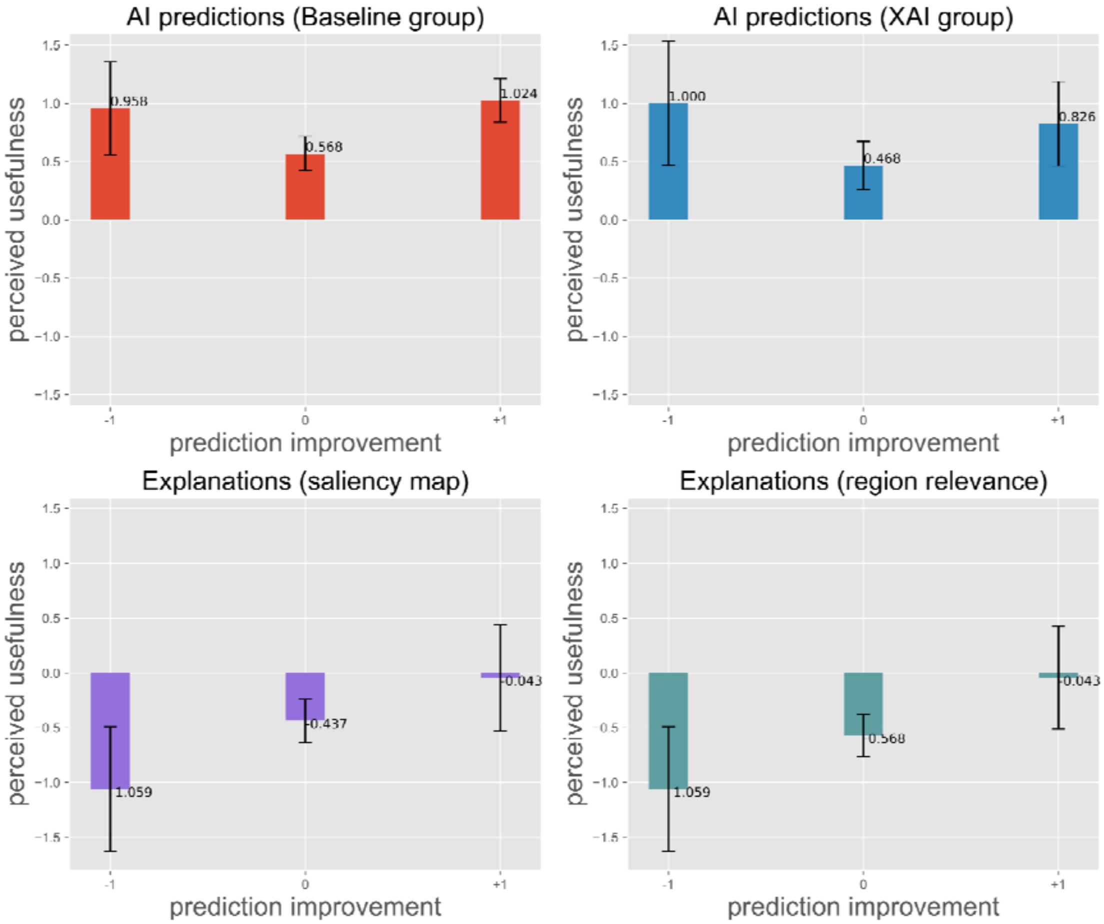


**Supplementary Figure 4**: The relationship between prediction improvement and participants' perceived usefulness ratings of each component. AI predictions in the Baseline and XAI groups (top), and explanations: saliency map (bottom left) and region relevance (bottom right).

medRxiv preprint doi: https://doi.org/10.1101/2025.06.08.25328588; this version posted June 9, 2025. The copyright holder for this preprint (which was not certified by peer review) is the author/funder, who has granted medRxiv a license to display the preprint in perpetuity.
This article is a US Government work. It is not subject to copyright under 17 USC 105 and is also made available for use under a CC0 license.

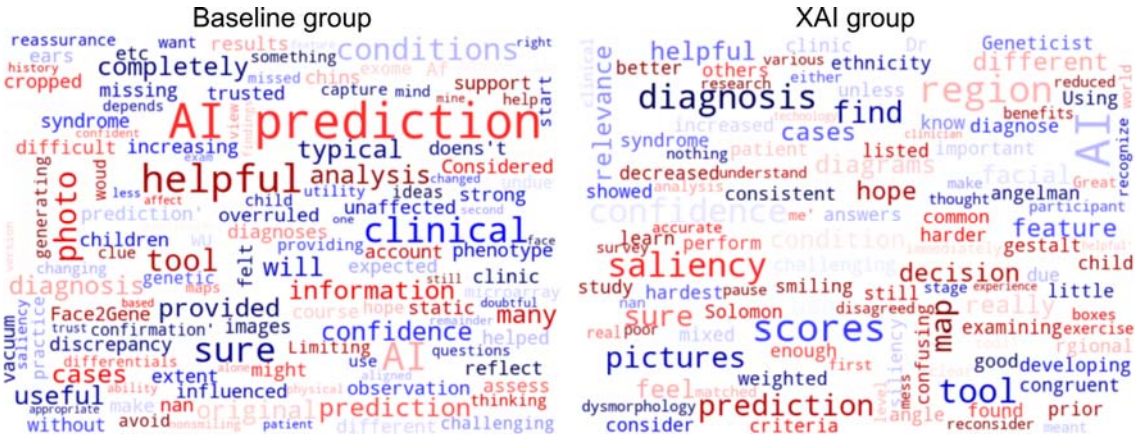


**Supplementary Figure 5**: Word cloud representations of free-text feedback from participants in the Baseline and XAI groups, illustrating the most frequently used terms.

medRxiv preprint doi: https://doi.org/10.1101/2025.06.08.25328588; this version posted June 9, 2025. The copyright holder for this preprint (which was not certified by peer review) is the author/funder, who has granted medRxiv a license to display the preprint in perpetuity. This article is a US Government work. It is not subject to copyright under 17 USC 105 and is also made available for use under a CC0 license.

**Supplementary Table 2**: Users' Qualitative Feedback on AI and XAI Support

| |
|---|
| **Baseline (AI-Only) Group** |
| AI overruled my clinical when there was a discrepancy. This doens't reflect how we make diagnoses in clinic where we have much more information to take into account rather than just an image. |
| Main observation was that it was difficult to assess some cases with static and/or cropped images (missing ears, chins, etc.). |
| AI prediction was a strong support to my original prediction |
| Considered the AI prediction to some extent when I felt sure it was a syndrome rather than unaffected. |
| The AI results mostly influenced me by decreasing or increasing my confidence in the a diagnosis. |
| I am not sure how much the AI predictions helped. Just like Face2Gene I wasn't sure I trusted AI that much. Of course this is in vacuum without any clinical information. In AfAm children AI might be more helpful as I think the typical phenotype can be different than expected and challenging. AI can be helpful in generating ideas but I woud hope we avoid an undue reliance in clinical practice This tool is not helpful. Limiting to 5 conditions when there are many more genetic conditions. Typical WU will start with exome or microarray which will capture most of these and other conditions. |
| I mostly use it if I have no clue what the child could have, or if I am thinking something but want confirmation |
| AI prediction was more useful in providing more confidence (reassurance) than completely changing mind (help to distinguish between differentials). I completely missed the saliency maps on my view. for the original questions - the utility of this depends on the particular patient (remainder of clinical history and other physical exam findings). |
| Useful tool. Some cases I was still doubtful |
| When AI prediction aligned with my diagnosis, it was helpful. However, when it was not, I did not trust the AI prediction over mine. 25In the second version of the photo (when the AI prediction answer was provided), I changed my answer to the one that the prediction provided. However, there were a few I am less confident on the prediction's ability. I also think a nonsmiling face with the right angle is most appropriate for analysis, and many of these photos did not have that feature which could affect the analysis of the tool. |
| When I wasn't sure based on the photo alone, the AI prediction was helpful. |
| **XAI Group** |

medRxiv preprint doi: https://doi.org/10.1101/2025.06.08.25328588; this version posted June 9, 2025. The copyright holder for this preprint (which was not certified by peer review) is the author/funder, who has granted medRxiv a license to display the preprint in perpetuity. This article is a US Government work. It is not subject to copyright under 17 USC 105 and is also made available for use under a CC0 license.

| |
|---|
| I did not find the saliency maps or rgional scores helpful. The gestalt is what is important.<br>I don't know how the AI does with ethnicity |
| When my decision was congruent with the AI tool it increased my confidence but when not it decreased my confidence on a diagnosis. The feature of the siliency and prediction were good but not the tool that weighted the facial regions. I found that angelman syndrome was<br>the hardest for me to diagnose and I was not very sure of the AI tools confidence. I hope I can get the answers and learn from this. |
| Did not consider AI prediction. Some cases harder than others due to smiling child or at different angle. I had little use of the saliency/region relevance scores in decision, unless they region scores are consistent enough to be listed at common criteria. |
| Using pictures is more challenging that examining a patient in the clinic. I feel some pictures mixed different features of the conditions which was confusing. I feel that AI is still developing. A prior study by Dr. Solomon showed that Geneticist perform better than AI for dysmorphology. |
| I did not find the map and region scores at all helpful. |
| not sure how to use this tool |
| My first thought is I hope I don't mess up your survey by being a poor research participant I really did not understand the AI diagrams and pictures either I recognize the condition immediately stage with that diagnosis or if the AI tool disagreed I reduced my confidence level and if this was the real world it would make me pause and reconsider am I really sure this is the diagnosis which as for the various boxes and diagrams they meant nothing to me |
| The AI prediction matched the clinical diagnosis in most of the cases. |
| saliency maps has no clear benefits to me |
| Great exercise on how much more accurate facial analysis technology is than an experience clinician (me) |
| I did not find the saliency or region relevance scores helpful |
